# 565. The Obesity Paradox in Relation to Acute Kidney Injury and Sepsis Development Following Burn Injury

**DOI:** 10.1093/jbcr/irag033.305

**Published:** 2026-04-06

**Authors:** Mbinui Ghogomu, Yves Balikosa, Ann Obi, Tsola Efejuku, Dominique Johnson, Steven E Wolf, Juquan Song, Amina El Ayadi

**Affiliations:** University of Texas Medical Branch at Galveston, Galveston, TX; University of Texas Medical Branch at Galveston, Galveston, TX; University of Texas Medical Branch at Galveston, Galveston, TX; University of Virginia, Charlottesville, VA; University of Texas Houston Health Science Center, Houston, TX; Division of Burn and Trauma Surgery, Department of Surgery, University of Texas Medical Branch, Galveston, Galveston, TX; University of Texas Medical Branch, Galveston, TX; Division of Burn and Trauma Surgery, Department of Surgery, University of Texas Medical Branch, Galveston, Galveston, TX

## Abstract

**Introduction:**

Although obesity is typically associated with adverse health outcomes, emerging evidence suggests paradoxical protective effects against mortality in burn and critically ill patients. However, most prior investigations were limited by small sample sizes. This study utilized a large, multicenter database to evaluate the effect of body mass index (BMI) on the development of acute kidney injury (AKI) and sepsis after severe burn injury.

**Methods:**

A retrospective analysis was performed using the TriNetX database. Adult burn patients (>18 years) were stratified by standardized BMI categories: Normal (20–24.9), Overweight (25–29.9), Obesity Class I (30–34.9), Obesity Class II (35–39.9), and Obesity Class III (≥40). Four matched cohorts were generated: Normal versus Overweight (5426 vs. 4610 pts), Normal versus Obesity Class I (5,426 vs. 5620 pts), Normal versus Obesity Class II (5426 vs. 3210 pts), and Normal versus Obesity Class III (5426 vs. 7163 pts). Cohorts were balanced for age, sex, race, and ethnicity. Outcomes of AKI and sepsis were assessed beginning one day post-injury.

**Results:**

Normal BMI patients had higher rates of sepsis and AKI compared with Overweight (sepsis: 9.95% vs. 6.03%, AKI: 7.28% vs. 4.39%, both *p*<.0001), Obesity Class I (sepsis: 9.70% vs. 5.50%, *p*=.0001; AKI: 6.72% vs. 4.74%, *p*=.0002), and Obesity Class II patients (sepsis: 10.13% vs. 4.00%, *p*<.0001; AKI: 6.62% vs. 4.59%, *p*=.0013). In contrast, Obesity Class III patients demonstrated higher complication risk, particularly AKI (sepsis: 10.82% vs. 9.77%, *p*=.1102; AKI: 15.29% vs. 11.98%, *p*=.0001).

**Conclusions:**

BMI significantly influences outcomes after burn injury. Overweight, Obesity Class I, and Obesity Class II patients exhibited a reduced risk of AKI and sepsis compared with normal BMI patients, supporting a potential obesity paradox. In contrast, Obesity Class III patients faced increased risk, particularly for renal complications.

**Applicability of Research to Practice:**

Incorporating BMI into burn care protocols may improve outcomes. Class III patients warrant early renal-protective strategies and tailored dosing, while normal BMI patients may benefit from enhanced nutritional support. Burn systems should integrate BMI into triage, resuscitation targets, and monitoring to optimize resource allocation.

**Funding for the study:**

This research was supported by an endowed fund for burn research and education, as well as by the Institute for Translational Sciences, supported in part by a Clinical and Translational Science Award from the National Center for Advancing Translational Sciences at the National Institutes of Health (NIH). The content of this study is solely the responsibility of the authors and does not necessarily represent the official views of the NIH.

